# Modular Dual Mobility Constructs Used for Recurrent Hip Instability

**DOI:** 10.7759/cureus.18251

**Published:** 2021-09-24

**Authors:** Andrew Yun, Marilena Qutami, Eric Carles

**Affiliations:** 1 Orthopaedic Surgery, Center for Hip and Knee Replacement, Providence Saint John's Health Center, Santa Monica, USA

**Keywords:** modular dual-mobility, dislocation, revision total hip arthroplasty, direct anterior approach

## Abstract

Background

Recurrent hip dislocation despite prior attempts at surgical stabilization is a dreadful and technically challenging complication. A modular dual mobility (MDM) articulation has shown promise in addressing this problem, which might seem intractable. Our purpose was to examine the outcomes of revision total hip arthroplasty (THA) with an MDM placed through a direct anterior (DA) approach when all other conservative and surgical treatments have failed.

Methods

Fifteen patients revised with an MDM for recurrent instability (RI) between 2012 and 2018 by a single surgeon at a single institution were reviewed retrospectively, with a minimum of two years' follow-up. All patients underwent full acetabular revision with an MDM articulation through a DA approach with intraoperative fluoroscopy. No stems were revised. Dislocations, complications, and clinical outcomes are reported.

Results

All patients had recurrent posterior instability with a mean number of 4 ± 2 (range: 2 to 8) dislocations prior to MDM revision THA (MDM rTHA). Eight patients had already failed surgical intervention for instability, and seven had failed repeated closed reductions and conservative care. After MDM rTHA, there were no dislocations at a mean follow-up of 4 ± 1 years (range: 2 to 8). Similarly, there were no further revisions or reoperations. Postoperatively, the mean cup inclination improved to 45 ± 2 degrees (range: 41 to 48), and the mean anteversion improved to 20 ± 2 degrees (range: 17 to 23). All cups were well-positioned utilizing fluoroscopic guidance. The mean effective head size increased from 32 mm to 44 mm. The mean hip disability and osteoarthritis disability score (HOOS, Jr) was 73 ± 25% (range: 40 to 100).

Conclusion

Refractory hip instability in THA may be effectively managed with an MDM articulation, even when prior attempts at surgical stabilization have failed. Intraoperative imaging and a direct anterior approach may aid the challenges of implant positioning and achieving hip stability in a revision setting.

## Introduction

A failed total hip arthroplasty (THA) due to recurrent dislocation is a dreadful complication, and the problem of multiple dislocations bears a physical and mental toll. Effective surgical treatment depends on the clarity of its causation, but the problem of recurrent instability is often multifactorial. In some cases, the etiology of dislocation can be attributed to issues of patient non-compliance, poor physiology, or technical error [1]. In others, the cause is inexplicable. Even technically well-positioned THAs have the potential to dislocate [2].

Numerous attempts to solve recurrent instability have met with limited success. The failure rate of historical treatments, including bracing, spica casting, modular exchange with larger heads, face shifting liners, soft tissue augmentation, and constrained liners, is reported up to 42.1% [3].

Recently, the invention and availability of a modular dual mobility (MDM) articulation have shown promise in addressing the problem, which seemed intractable. Multiple series have shown a statistically significant reduction in dislocations in the primary and revision THA (rTHA) compared to conventional fixed bearing designs [4,5].

In this study, we present our limited experience with the problem of refractory hip instability. We hypothesized that an MDM articulation placed through a direct anterior (DA) approach with intraoperative imaging could potentially mitigate the risk of future dislocation. We theorized that the triad of improved mechanical stability in the dual articulation, a deliberate preservation of posterior pseudocapsule, and guided placement of implants in the “safe zone” would be protective. According to the Lewinnek definition, the safe zone for cups to minimize the risk of dislocation is between 30 and 50 degrees of inclination and 10 to 30 degrees of anteversion [6]. This study examines the technical details, clinical outcomes, radiographic metrics, and complications with the direct anterior technique in this challenging population.

## Materials and methods

We retrospectively reviewed 15 consecutive patients who underwent MDM rTHA for recurrent instability between 2012 and 2018. A DA approach with intraoperative fluoroscopy was used in all cases. Surgery was performed by a single surgeon at a single institution with a minimum of two years' follow-up. All patients had previously experienced two or more dislocations (range: two to eight). The indication for revision surgery was either the failed revision THA or the failed conservative treatment with recurrent dislocations. Only patients with acetabular cup, liner, and femoral head exchange were included. Patients treated with a modular head exchange or isolated liner exchange into a retained shell during their revision surgeries were excluded. Also, patients with recurrent instability treated with femoral stem revision alone or in combination with acetabular cup revision were excluded. Charts were reviewed for indications, clinical history, and prior treatment failures. The primary outcome measures were dislocation after MDM rTHA and the need for subsequent rTHA in case of further instability. Secondarily, records were reviewed for complications, reoperations, and readmissions. Clinical outcomes measured were revision and the hip disability and osteoarthritis disability score (HOOS, Jr). The HOOS is a validated patient reported outcome measure of hip function [7]. The study was approved by the Providence Saint Joseph Health Institutional Review Board (STUDY2020000262).

Surgical technique

All patients were placed on the Hana (Mizuho OSI; Union City, CA) table for a DA approach. Patients with an irreducible dislocation underwent an attempt at closed reduction with gentle traction and external rotation after induction of adequate anesthesia. Closed reduction was attempted to restore the characteristic anatomic landmarks prior to incision.

A DA approach was performed as described by Matta [8]. Dissection was taken to the hip level in the standard fashion. After an H-shaped capsulotomy with preservation of all capsular tissue, an examination of impingement and direction of instability through a range of motion (ROM) was performed. Notably, the leg was temporarily freed from the Hana table for accurate testing.

Once the modular head was removed in the standard fashion, the stem was inspected for stability, position, version, and trunnion condition. As previously mentioned, only those hips with well-fixed and well-positioned stems were included in this analysis.

**Figure 1:**
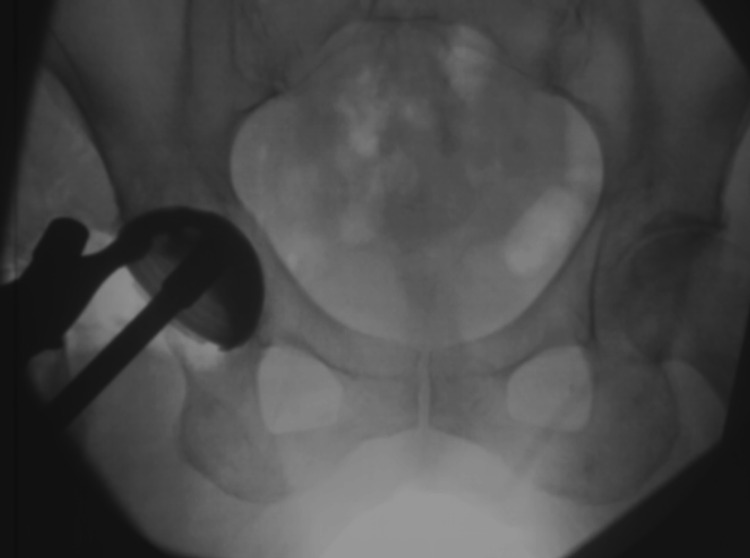
Intraoperative image to guide acetabular cup placement. The intraoperative image during right cup insertion indicates that the stem is displaced posteriorly.

The acetabulum was then exposed by rotating the leg externally and displacing the femoral trunnion posteriorly. In all cases in this series, the prior acetabular component was removed either for implant malposition or for incompatibility with the chosen dual mobility articulation (Modular Dual Mobility; Stryker Kalamazoo, MI). An acetabular explantation system (Explant Acetabular Cup Removal System; Zimmer Warsaw, IN) was used according to the known size of the cup. If the cup size was unknown, a measurement of the cup’s diameter was calculated based on a calibrated digital radiograph. Once the cup was removed, the remaining bone of the acetabulum was inspected for defects, and the acetabulum was spherically reamed under fluoroscopic guidance. A modular titanium shell (Trident II; Stryker Kalamazoo, MI) was then placed using fluoroscopic imaging to guide abduction and anteversion (Figure 1). Adjunctive screws were used as needed for stability. The cobalt chrome liner was then impacted into the titanium shell. A trial dual mobility head was placed on the stem, and the hip was reduced. The trials were clinically tested for stability, range of motion, and impingement, using the same testing technique noted previously. Intraoperative fluoroscopy was used to assess leg length, offset, and implant position. Adjustments to either improve stability or decrease impingement were then made before the real implants were placed. The capsule was formally repaired by re-approximating the capsular leaves at the end of the procedure. 

Radiographic measurement

Preoperatively, radiographs were evaluated for direction of instability, anteversion, cup inclination, and leg length discrepancy. Postoperatively, radiographs were evaluated for anteversion, cup inclination and leg length discrepancy. Anteversion was determined from the ellipse formed by the opening of the cup using the Radlink software (El Segundo, CA). Leg length discrepancy was determined by the vertical distance from a similar point on the lesser trochanters to a horizontal line drawn across the bottom of each radiographic teardrop on anteroposterior (AP) radiograph of the pelvis. The implants were evaluated for loosening, migration, and radiolucent lines, as previously well-described.

Patient management

In this series, patients were allowed to progress with weight-bearing as tolerated and without further dislocation precautions. Patients were anticoagulated with aspirin for three weeks unless stronger anticoagulation was specifically indicated based on risk stratification, degree of immobility, and prior medical history.

## Results

Patients included 11 women and four men. There were nine right and six left MDM rTHAs. The mean age of patients at the time of MDM rTHA was 71 ± 10 years (range: 54 to 89). The mean time to follow-up was 4 ± 1 years (range: 2 to 8). Initially, 13 patients had a posterior approach for the primary hip replacement, and two patients had an anterior approach for the primary hip replacement. All 15 patients had recurrent posterior dislocations.

**Figure 2:**
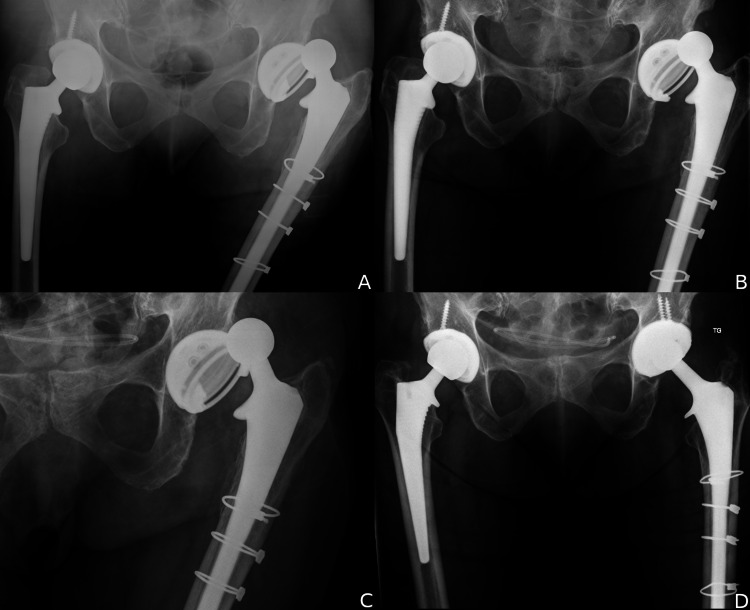
Recurrent instability in a patient with multiple prior attempts at surgical stabilization. (A) Dislocation despite prior stem revision two years earlier and a constrained liner one year earlier. (B) A second failed constrained liner with dislocation one year later. (C) A third failed constrained liner two years later. (D) No further dislocations at six years with a dual mobility bearing.

The mean number of dislocations prior to MDM rTHA was 4 ± 2 (range: 2 to 8). Seven patients had failed conservative treatment with either posterior precautions, physical therapy, hip bracing, or spica casting. In contrast, eight patients had already undergone prior rTHA specifically for instability. Of these, five had one failed additional surgery, two had two failed additional surgeries, and one had four additional failed surgeries. Notably, two of these patients had a failed attempt at stabilization with constrained liners. One patient with four failed prior surgeries had sequentially undergone a 1) stem revision with an extended trochanteric osteotomy for a retroverted stem, 2) first constrained liner, 3) second constrained liner, and 4) third constrained liner before conversion to the MDM construct (Figure 2). The second of these two patients had two prior rTHAs and had a failed large head modular exchange, followed by a failed constrained liner.

Clinical outcomes

After MDM rTHA, there were no further dislocations in the 15 hips at a minimum of two years' follow-up. There were no further revisions or reoperations. The mean HOOS, Jr score was 73 ± 26 (range: 40 to 100). Six years after MDM rTHA, the death of an 88-year-old woman was reported, due to causes unrelated to the revision surgery.

At the time of MDM rTHA, the mean estimated blood loss (EBL) was 258 ± 126 mL (range: 100 to 500). The explantation system was used in all cases without clinically significant bone loss. Preoperatively, the mean cup size was 52 ± 4 mm (range: 46 to 60), and the mean head size was 32 ± 2 mm (range: 28 to 36). Postoperatively, the mean cup size increased to 56 ± 4 mm (range: 48 to 62). The mean effective head size of the polyethylene ball increased to 42 ± 4 mm (range: 36 to 48). A cementless, titanium, hemispherical acetabular shell (Trident II; Stryker Kalamazoo, MI) was used in all cases. Twelve cups were stable without screws, and three cups required one screw for adjunctive fixation. No stems were revised nor found to be malpositioned. There were no reported intraoperative complications.

The mean length of stay was 3 ± 2 days (range: 1 to 6). Thirteen patients were discharged to home, and two patients were discharged to a skilled nursing facility. 

Radiographic outcomes

**Figure 3:**
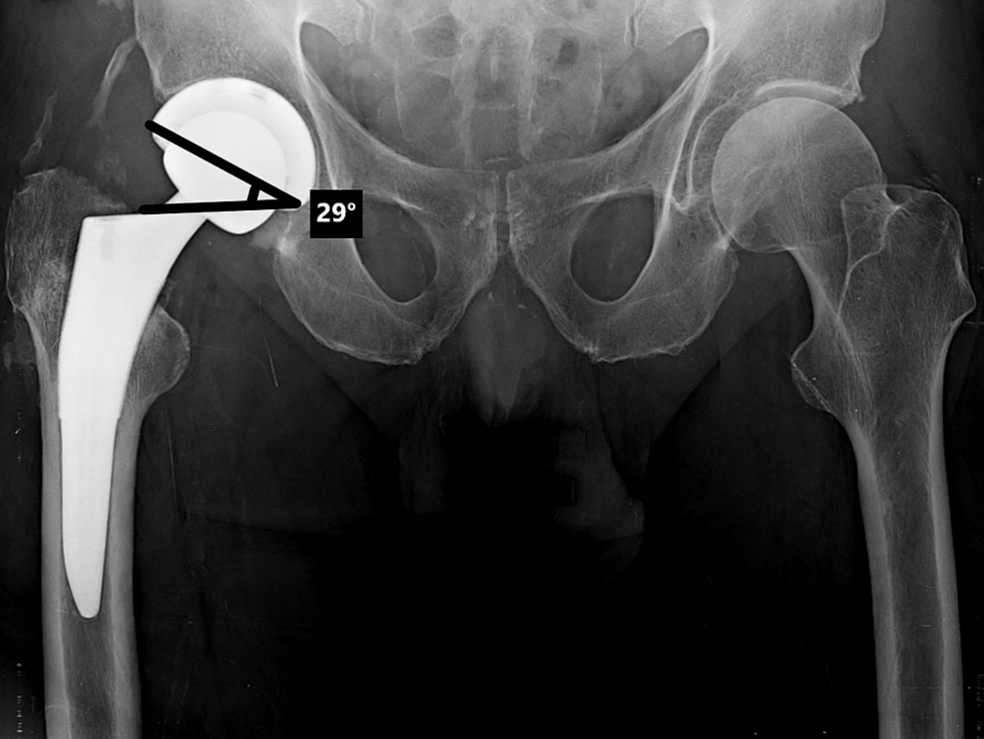
AP radiograph in a patient with recurrent instability. The cup inclination of 29 degrees is outside the so-called safe zone.

Preoperatively, the mean cup abduction angle was 46 ± 8 degrees (range: 29 to 62), and the mean anteversion was 15 ± 4 degrees (range: 7 to 21). Five of these (33%) were outside the acknowledged safe zone (Figure 3) [6]. Postoperatively, the mean cup abduction angle was 45 ± 2 degrees (range: 41 to 48), and the mean anteversion was 20 ± 2 degrees (range: 17 to 21). None were outside the Lewinnek safe zone. For leg length inequality, the mean amount of preoperative discrepancy was 2 ± 1 mm (range: 0 to 4). The mean amount of postoperative leg length discrepancy was 2 ± 3 mm (range: 0 to 13). The patient with a 13 mm leg length discrepancy was intentionally lengthened to maximize soft tissue tension after four prior failed rTHAs. At follow-up, all hips were radiographically stable.

## Discussion

Failed treatment of recurrent instability can be discouraging. We turned to the use of MDM implants shortly after they had gained FDA approval in 2009. Despite our initial skepticism, we explored this option out of desperation when all prior attempts with surgical treatment had failed. Specifically, among our first cases was a salvage attempt at stabilization in a patient who continued to dislocate despite multiple failed constrained liners. To our surprise and relief, the patient has not dislocated in six years of follow-up.

To our knowledge, there are no published studies of an MDM with a DA approach in revision THA. We hypothesized that we could decrease the probability of future dislocations by 1) improving the intrinsic mechanical stability with a larger head MDM articulation, 2) removing the existing cup with minimal bone loss, 3) placing the new components as accurately as possible within the safe zone to maximize the effective ROM, and 4) leveraging an anterior approach to the hip that comparably reduces the risk of posterior dislocation. 

While we appreciate the controversy surrounding an optimal approach in THA, we also recognize the relative difference in rates of instability between the posterior and anterior approaches. We, therefore, deliberately approached the hip joint anteriorly. As all the patients in our cohort dislocated posteriorly, we hypothesized that limiting further dissection in the posterior tissues would be less destabilizing. In two studies using an MDM with the posterior approach, Hartzler et al. reported a recurrent dislocation rate of 3% [9], and Plummer et al. reported a rate of 2.7% [10]. We concede that these tissue-preserving benefits of the DA approach in rTHA for instability are merely theoretical and warrant further investigation in comparative studies.

The mechanical advantages of the dual mobility articulation in improving stability are well-described and also well-documented. Bosquet et al. theorized that an increased effective head size of the polyethylene ball would improve the head-to-neck ratio, reduce component-component impingement, and improve the jump distance [7,11]. Similarly, the mean increase in effective head size from 32 mm to 44 mm in this series may be largely responsible for the reduction in postoperative instability. Leveraging the dual articulation, Hartzler et al. have confirmed that the rate of dislocation in revision with an MDM was significantly lower than the rate with fixed bearing large heads [9]. More broadly, the success of dual mobility bearings has been confirmed by the Swedish Registry, the United Kingdom Registry, and a United States multi-center study, with dislocation rates as low as 2% [12-14].

We have expanded the indications and the use of MDM through a DA approach for recurrent instability. Consequently, we have since abandoned modular fixed bearing head exchange and constrained liners in all but the most extreme cases of abductor or soft tissue deficiency. Compared to the reported dislocation rate of 16% to 18% with a constrained liner, our results with an MDM are far more favorable [7,15]. We also noted that two patients in our series who had failed prior attempts with a constrained liner did not experience any further dislocations. Similarly, Plummer et al. reported that three patients were treated successfully with an MDM after failing prior constrained liners [10].

**Figure 4:**
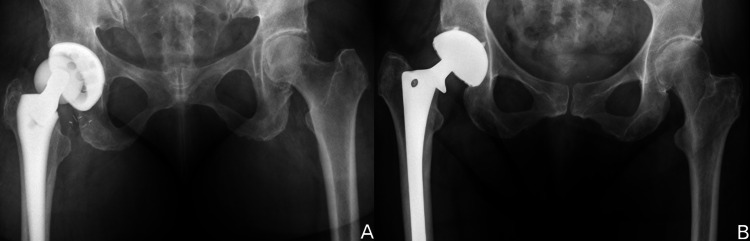
Patients with recurrent instability related to implant malposition. (A) AP radiograph of a vertically malpositioned cup. (B) AP radiograph of a retroverted cup with asymmetric bearing wear.

**Figure 5:**
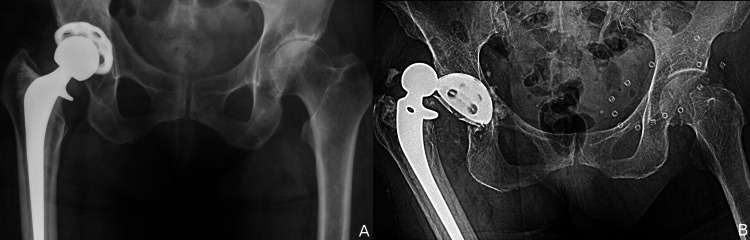
Older acetabular implants with locking mechanisms incompatible with a dual mobility liner required removal. (A) AP radiograph of a dislocating patient with a Duraloc Bantam cup (DePuy). (B) AP radiograph of a dislocating patient with an HG 2 cup (Zimmer).

All cases in this series required removal of the preexisting acetabular shell either for malposition (33%) (Figure 4) or for an incompatible locking mechanism (67%) (Figure 5). The difficulty and morbidity of removing a well-fixed cup were greatly reduced with the explantation system, as previously reported. Visualization for cup removal through a DA approach was straightforward. Bone loss was minimal, and 80% of cups did not require further stabilization with screw fixation. For the ones that required screws, we found the presence of screw holes in the modular shell to be advantageous. Alternative monoblock, non-modular dual mobility designs are not amenable to supplemental screw fixation.

**Figure 6:**
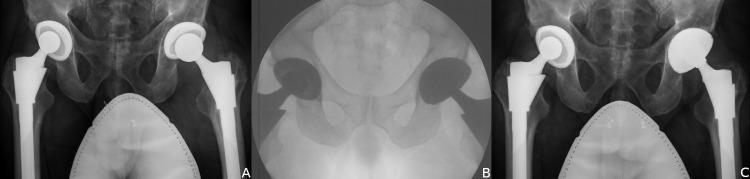
Management of left hip recurrent dislocation. (A) AP radiograph in a patient with recurrent dislocation despite prior revision with a modular head exchange. (B) Intraoperative fluoroscopy confirming cup orientation, offset and leg length. (C) AP radiograph three years after MDM revision with no further dislocations.

Proper positioning of the acetabulum is especially important in the management of recurrent dislocation. Cup malposition is poorly tolerated, even despite the increased mechanical stability of an MDM. An editorial by Horriat and Haddad argued that the “biomechanical advantage of dual mobility bearings should not create the wrong impression that it can compensate for a lack of surgical skill” [16]. Given the distorted anatomy and scarring that compromise visualization in rTHA, the best attempts at an accurate placement of the cup by direct visualization are impeded by the lack of anatomic landmarks. Rather, we found that fluoroscopic imaging provided valuable real-time guidance for intraoperative adjustments of cup orientation. In the salvage of these difficult cases with recurrent instability, cups were placed with minimal variance, with a mean inclination of 45 degrees and an anteversion of 20 degrees. We also found that intraoperative imaging offered a reliable way to calculate and choose the head length. With the trials in place and the hip reduced, fluoroscopy was used to confirm that sufficient hip length and offset were restored to maintain soft tissue tension (Figure 6).

The major limitation of this study is that it is a single surgeon’s small retrospective series. There is an intrinsic risk of bias with a single surgeon, so we have tried to rely on objective points of reference, such as dislocations, patient-reported outcomes, and radiographic metrics. With the limited sample pool of only 15 patients, it is possible that a larger sample population of patients may reveal a complication profile similar to that of other series. Another major limitation is the lack of a control group for comparison. While we have attempted to compare our findings with other reported series of the MDM using a posterior approach, a direct comparison of our own using the MDM with a different surgical approach or a different indication for surgery could serve as a control. Alternatively, a cohort of patients with refractory instability managed with a modular head exchange or a constrained liner using the DA approach could be compared. We will recognize these comparisons as a possibility for future study and their absence as a limitation of the current study. Finally, we acknowledge that this small MDM series is, at best, a narrow contribution with limitations. However, it describes a novel arthroplasty technique that addresses the exceptionally difficult challenge of refractory hip instability. In this regard, it adds to the existing but limited body of literature regarding options for surgical treatment in this high-risk patient population.

## Conclusions

In conclusion, revision THA with a modular dual mobility construct may be an effective solution for recurrent, intractable instability. In our limited series, we found no further dislocations after revising to an MDM construct, even in patients who had failed prior attempts at surgical correction. The use of a DA approach with formal capsular repair may be protective against further hip instability, and the anterior approach does not impede visualization. Intraoperative feedback from fluoroscopic imaging may assist with targeted implant placement and decreasing positional variance. Further studies are warranted to confirm these findings.
